# Immunofluorescence-Based Assay for High-Throughput Analysis of Multidrug Resistance Markers in Non-Small Cell Lung Carcinoma Patient-Derived Cells

**DOI:** 10.3390/diagnostics13243617

**Published:** 2023-12-07

**Authors:** Jelena Dinić, Ana Podolski-Renić, Miodrag Dragoj, Sofija Jovanović Stojanov, Ana Stepanović, Ema Lupšić, Milica Pajović, Mirna Jovanović, Dušica Petrović Rodić, Dragana Marić, Maja Ercegovac, Milica Pešić

**Affiliations:** 1 Department of Neurobiology, Institute for Biological Research “Siniša Stanković”—National Institute of the Republic of Serbia, University of Belgrade, Bulevar Despota Stefana 142, 11108 Belgrade, Serbia; ana.podolski@ibiss.bg.ac.rs (A.P.-R.); miodrag.dragoj@ibiss.bg.ac.rs (M.D.); sofija.jovanovic@ibiss.bg.ac.rs (S.J.S.); ana.kostic@ibiss.bg.ac.rs (A.S.); ema.lupsic@ibiss.bg.ac.rs (E.L.); milica.pajovic@ibiss.bg.ac.rs (M.P.); mirna.jovanovic@ibiss.bg.ac.rs (M.J.); 2 Department of Thoracic Pathology, Clinical Center of Serbia, Service of Pathohistology, University of Belgrade, Pasterova 2, 11000 Belgrade, Serbia; 1980dusica@gmail.com; 3 Clinic for Pulmonology, Faculty of Medicine, University of Belgrade, Dr Koste Todorovića 26, 11000 Belgrade, Serbia; draganamaric23@yahoo.com; 4 Clinic for Thoracic Surgery, Faculty of Medicine, University of Belgrade, Pasterova 2, 11000 Belgrade, Serbia; majaerce@verat.net

**Keywords:** lung cancer, NSCLC, multidrug resistance, MDR markers, ABCB1, ABCC1, ABCG2, immunofluorescence assay, primary cell cultures

## Abstract

Lung cancer remains the leading cause of cancer death globally, with non-small cell lung cancer (NSCLC) accounting for the majority of cases. Multidrug resistance (MDR), often caused by ATP-binding cassette (ABC) transporters, represents a significant obstacle in the treatment of NSCLC. While genetic profiling has an important role in personalized therapy, functional assays that measure cellular responses to drugs are gaining in importance. We developed an automated microplate-based immunofluorescence assay for the evaluation of MDR markers ABCB1, ABCC1, and ABCG2 in cells obtained from NSCLC patients through high-content imaging and image analysis, as part of a functional diagnostic approach. This assay effectively discriminated cancer from non-cancer cells within mixed cultures, which is vital for accurate assessment of changes in MDR marker expression in different cell populations in response to anticancer drugs. Validation was performed using established drug-sensitive (NCI-H460) and drug-resistant (NCI-H460/R) NSCLC cell lines, demonstrating the assay’s capacity to distinguish and evaluate different MDR profiles. The obtained results revealed wide-ranging effects of various chemotherapeutic agents on MDR marker expression in different patient-derived NSCLC cultures, emphasizing the need for MDR diagnostics in NSCLC. In addition to being a valuable tool for assessing drug effects on MDR markers in different cell populations, the assay can complement genetic profiling to optimize treatment. Further assay adaptations may extend its application to other cancer types, improving treatment efficacy while minimizing the development of resistance.

## 1. Introduction

Lung cancer is the leading cause of cancer-related deaths worldwide. Non-small cell lung carcinoma (NSCLC) is the most common form of lung cancer and accounts for nearly 80% of all lung cancers [1]. A major cause of treatment failure in NSCLC is the development of resistance to therapy, often caused by overexpression of ATP-binding cassette (ABC) transporters [2]. These membrane transporters, which include ABCB1 (multidrug resistance protein or P-glycoprotein, MDR1/P-gp), ABCC1 (multidrug resistance-associated protein 1, MRP1), and ABCG2 (breast cancer resistance protein, BCRP), play an essential role in mediating drug efflux [3]. They function on both cell and vesicle membranes, limiting cancer cell exposure to therapeutics and contributing to multidrug resistance (MDR) [3]. Different ABC transporters can recognize and transport specific substrates, and their substrate specificity can vary widely. ABCB1 substrates include various chemotherapeutics such as cisplatin, vinorelbine, paclitaxel, etoposide, and docetaxel [4,5]. ABCC1 extrudes etoposide, paclitaxel, and vinorelbine, while ABCG2 is involved with the efflux of docetaxel [5]. Regardless of their substrate status, specific drugs also have the ability to impact the expression of ABC transporters. For instance, paclitaxel, doxorubicin, and gemcitabine have been shown to impact the mRNA expression levels of ABCB1, ABCG2, and ABCC1 [6]. Carboplatin has been associated with inducing drug resistance mediated by ABCB1 [7] and ABCG2 expression [8]. Additionally, cisplatin has been found to up-regulate the expression of ABCC1 and ABCC2 [6], while etoposide treatment can induce the expression of ABCB1 and ABCG2 in lung cancer cells [9]. Increased ABC transporter expression is associated with unfavorable treatment responses leading to reduced survival rates, impacting not only NSCLC but also other tumor types such as glioblastoma, neuroblastoma, and prostate, breast, renal, and thyroid cancers [2]. Therefore, in the era of the development of personalized cancer treatments, special efforts should be made to detect and overcome MDR. Although NSCLC has been found to be intrinsically multidrug-resistant, mainly due to the overexpression of ABC family proteins, acquired resistance is also a major obstacle to successful treatment [10,11]. In particular, significant activation of ABCB1 expression has been observed during chemotherapy [10]. Improvements in drug development as well as techniques to detect various MDR markers are urgently needed to optimize therapy for NSCLC patients.

Although advances in sequencing technology and target identification support the implementation of personalized therapy, only 3–9% of cancer patients show a satisfactory response to treatment [12]. Functional assays can help identify the most effective treatments for individual patients by measuring the functional response of cells or tissues to drugs or other stimuli. They can also help predict treatment response and resistance, optimize drug combinations, and reduce the risk of adverse effects [13]. Recently, a functional diagnostics approach was proposed that combines the response of patient-derived cancer cells to treatment with the patient’s genetic profile to recommend the optimal therapy. Starting from pharmacological screening ex vivo, in contrast to conventional diagnostics, functional diagnostics can be used to identify the sensitivity of patient-derived cancer cells that cannot be detected by sequencing [12]. Although mutation status has been used widely to predict the response to targeted therapy, it cannot guarantee whether the patient will respond or not. In addition, functional diagnostics can test a patient’s response to multiple drugs at once and identify drugs that induce MDR. Together with routine screening of appropriate molecular targets, such an approach could provide clinical oncologists with valuable information on which therapeutics should be used in clinical practice.

High-content imaging involves the use of automated microscopy to visualize, characterize, and quantify the effects of therapeutics on cell populations, producing useful data sets for further analysis. A number of automated microplate-based cell imaging cytometers have become available in recent years, such as the CellInsight CX series (Thermo Fisher Scientific, Waltham, MA, USA) [14], Spark Cyto (Tecan, Männedorf, Switzerland) [15], Celigo (Nexcelom Bioscience, Lawrence, MA, USA) [16], IN Cell Analyzer 2200 (GE, Buckinghamshire, UK) [17], CELLAVISTA 4 (Synentec, Elmshorn, Germany) [18], Cytation 5 (BioTek, Winooski, VT, USA) [15], and ImageXpress Pico (Molecular Devices, San Jose, CA, USA) [19,20]. These automated microplate-based cell imaging cytometers allow for the analysis of live and dead cells and various targets of interest within the same cells, reducing the number of experimental steps that could affect assay robustness. The main benefit of image cytometry compared to other methods such as Western blot is the ability to successfully overcome the issue of heterogeneity and collect data from different cell populations without averaging signal intensities.

Our goal was to develop an immunofluorescence-based in vitro assay to assess the expression of MDR markers that can be used to analyze NSCLC patient-derived cells as part of a functional diagnostics approach. A key feature of this assay is that it distinguishes the MDR profile of cancer and non-cancer cells in mixed cell cultures. To validate the assay, the treatments were performed with eight classic chemotherapeutic agents that can affect the expression of MDR markers.

## 2. Materials and Methods

### 2.1. The Establishment of Primary Cultures from NSCLC Tissue Samples

NSCLC samples were collected from the Clinic for Thoracic Surgery at the Clinical Center of Serbia after obtaining informed consent from the patients and approval from the Ethics Committee of the Clinical Center of Serbia (ref. number 623/4). The patients did not receive any drug treatment before surgery. Tissue samples were collected during surgery, and the NSCLC diagnosis, histological grade, stage, necrosis, and lymph node invasion status were determined by histopathological analysis. The histological grades of the collected NSCLC samples are as follows: TR58 (grade IIA), TR64 (grade IIIA), TR65 (grade IIIA), TR84 (grade IB), and TR87 (grade IIB). An EGFR L858R mutation was identified in sample TR64 by the Cobas EGFR Mutation Test v2 (Hoffman-La Roche Ltd., Basel, Switzerland). The surgical specimens were placed in a sterile tube containing antibiotic-antimycotic solution (Sigma-Aldrich Chemie GmbH, Taufkirchen, Germany) and immediately transported to the research laboratory for further processing.

Upon arrival, the tissue was manually chopped with a surgical blade in a Petri dish under sterile conditions. The samples were cut into 3–5 mm pieces and further dissociated using the Tumor Dissociation Kit (Miltenyi Biotec, Bergisch Gladbach, Germany). The Tumor Dissociation Kit was used to dissociate the cells from the tumor tissue according to the manufacturer’s instructions. The samples were incubated in a 37 °C orbital shaker (KS 4000 ic control, IKA, Königswinter, Germany) at 300 rpm for 90 min. After incubation, the dissociated tissue was placed in DMEM/Ham’s F12 (1:3 ratio) growth medium supplemented with 5% fetal bovine serum (Corning, NY, USA), antibiotic-antimycotic solution, 4 µg/mL hydrocortisone (Sigma-Aldrich Chemie GmbH, Taufkirchen, Germany), 1 µg/mL insulin (Sigma-Aldrich Chemie GmbH, Taufkirchen, Germany), 10 ng/mL epidermal growth factor (BioLegend, San Diego, CA, USA), and 24 µg/mL adenine (Sigma-Aldrich Chemie GmbH, Taufkirchen, Germany). The DMEM and Ham’s F12 growth media were purchased from Sigma-Aldrich Chemie GmbH (Taufkirchen, Germany).

The dissociated tissue was cultured in T-25 cell culture flasks until cell attachment was observed before the medium was replaced. If no cell attachment was achieved within 7 days, the sample was discarded. Successful patient-derived cultures were maintained at 37 °C in a humidified atmosphere with 5% CO_2_ and grown to confluence before undergoing further experiments. If more than 50% of the primary cells exhibited fibroblast-like morphology, partial trypsinization was performed to enrich the culture with NSCLC-like cells. Partial trypsinization was performed by detaching the fibroblast-like cells with 1 mL of 0.05% trypsin for 1 min at 37 °C, followed by 2 min at room temperature (RT).

### 2.2. Cell Lines

Human non-small cell lung carcinoma cells (NCI-H460) were purchased from the American Type Culture Collection (Rockville, MD, USA), and normal embryonic lung fibroblasts (MRC-5) were purchased from the European Collection of Authenticated Cell Cultures (Salisbury, UK). Multidrug-resistant NCI-H460/R cells overexpressing ABCB1 were selected from the NCI-H460 cells after selective pressure with doxorubicin [21]. All the cell lines were maintained in MEM medium (Sigma-Aldrich Chemie GmbH, Taufkirchen, Germany) supplemented with 10% FBS, 2 mM L-glutamine, and antibiotic-antimycotic solution. All the cell lines were maintained at 37 °C in a humidified atmosphere with 5% CO_2_.

### 2.3. Drugs and Treatments

Vinorelbine and pemetrexed were purchased from Selleckchem (Houston, TX, USA). Carboplatin, cisplatin, docetaxel, etoposide, gemcitabine, gefitinib, and paclitaxel were purchased from Sigma-Aldrich Chemie GmbH (Taufkirchen, Germany). The cisplatin, carboplatin, and gemcitabine were dissolved in sterile water, while the etoposide, docetaxel, vinorelbine, paclitaxel, gefitinib, and pemetrexed were dissolved in DMSO and stored at −20 °C. Before treatment, all the stock solutions were freshly diluted in sterile water. The following concentrations were used for the treatments: vinorelbine (100, 250, 500, 750, and 1000 nM); pemetrexed (50, 75, 100, 200, and 300 µM); carboplatin (10, 25, 50, 75, and 100 µM); cisplatin (5, 7.5, 10, 12.5, and 15 µM); docetaxel (1, 2, 3, 4, and 5 µM); etoposide (10, 15, 20, 25, and 30 µM); gemcitabine (10, 25, 50, 75, and 100 µM); paclitaxel (1, 2, 3, 4, and 5 µM); gefitinib (50, 100, 200, 300, and 400 nM).

The primary cells were seeded in black, clear bottom 384-well cell culture microplates (Thermo Fisher Scientific, Waltham, MA, USA) in 50 µL of cell growth medium at a density of 1000 cells per well. The compounds were administered 72 h after seeding. The treatment lasted 7 days.

The NCI-H460 and NCI-H460/R cell lines were seeded in black, clear bottom 384-well cell culture microplates (Thermo Fisher Scientific, Waltham, MA, USA) in 50 µL of MEM medium at a density of 1000 cells per well and in co-culture with MRC-5 at a ratio of 1:1. The compounds were administered 48 h after seeding. The treatment lasted 24 h.

### 2.4. Immunofluorescence Assay

The immunofluorescence assay for the quantification of MDR markers was optimized to distinguish cancer cells from stromal cells using a cytokeratin 8/18 (CK8/18) antibody cocktail. The cells were fixed with 4% paraformaldehyde for 20 min at RT and washed three times with PBS using the Wellwash Versa microplate washer (Thermo Fisher Scientific, Waltham, MA, USA). The cells were then blocked with 2% bovine serum albumin (BSA) in PBS for 1 h at RT. The anti-CK8/18 primary antibody cocktail (clone SU0338, #MA5-32118, Thermo Fisher Scientific, Waltham, MA, USA) was diluted 1:1000 in 2% BSA in PBS and incubated with the cells at 4 °C overnight. Co-immunostaining with antibodies against MDR markers was performed to detect the presence of ABCB1, ABCC1, and ABCG2 in both the cancer and stromal cells. The anti-ABCB1 monoclonal antibody (clone C219, #MA1-26528, Thermo Fisher Scientific, Waltham, MA, USA) was diluted 1:100 in 2% BSA in PBS and incubated with the cells at 4 °C overnight. Anti-ABCC1 monoclonal antibody (clone IU5C1, #MA5-16079, Thermo Fisher Scientific, Waltham, MA, USA) and anti-ABCG2 monoclonal antibody (clone 1H2, #ab130244, Abcam, Cambridge, UK) were diluted 1:1000 in 2% BSA in PBS and incubated with the cells at 4 °C overnight. The cells were washed three times with PBS using the microplate washer before adding Alexa Fluor 555 goat anti-mouse secondary antibody (#A-21422, Thermo Fisher Scientific, Waltham, MA, USA) and Alexa Fluor 488 goat anti-rabbit secondary antibody (#A-11008, Thermo Fisher Scientific, Waltham, MA, USA) diluted 1:1000 in 2% BSA in PBS. The secondary antibodies were incubated with the cells at RT for 2 h in the dark. To label the nuclei, the cells were incubated for 2 h in the dark with Hoechst 33342 at a final concentration of 1 µg/mL at RT. Finally, the cells were washed three times with PBS and stored at 4 °C in the dark before imaging.

The fluorescently labeled cells were imaged using the ImageXpress Pico Automated Cell Imaging System (Molecular Devices, San Jose, CA, USA) with a 4x objective after determining the appropriate exposure time for each illumination filter. Analysis of the obtained images was performed using CellReporterXpress software v. 2.8.2.669 (Molecular Devices). The cytotoxicity of the compounds was determined using the Cell Scoring Analysis Protocol. Briefly, to ensure accurate segmentation of nuclear Hoechst 33342 and cytoplasmic CK8/18 staining, the minimum and maximum widths for the nucleus and whole cell, as well as the correct signal intensity thresholds, were determined. Analysis revealed the total number of cells (Hoechst 33342-positive cells), the number of cancer cells (Hoechst 33342-positive and CK8/18-positive cells), and the number of stromal cells (Hoechst 33342-positive and CK8/18-negative cells) in each microplate well. Expression of MDR markers was determined using the Multi-Wavelength Cell Scoring Analysis Protocol. Briefly, to ensure accurate segmentation of the nuclear Hoechst 33342 and cytoplasmic staining (CK8/18, ABCB1, ABCC1, ABCG2), the minimum and maximum widths for the nucleus and whole cell, as well as the correct signal intensity thresholds, were determined. Co-staining of ABCB1, ABCC1, and ABCG2 with CK8/18 and Hoechst 33342 allowed identification and quantification of MDR markers in the cancer cells (Hoechst 33342-positive, CK8/18-positive, and MDR marker-positive cells), and stromal cells (Hoechst 33342-positive, CK8/18-negative, and MDR marker-positive cells) in each microplate well.

### 2.5. Statistical Analysis

Statistical analysis was performed by GraphPad Prism software v. 8.0.2 (San Diego, CA, USA). The results were analyzed by two-way analysis of variance (ANOVA) and Dunnett’s multiple comparisons test, and the accepted level of significance was *p* < 0.05.

## 3. Results and Discussion

### 3.1. Immunoassay Principle

**Figure 1:**
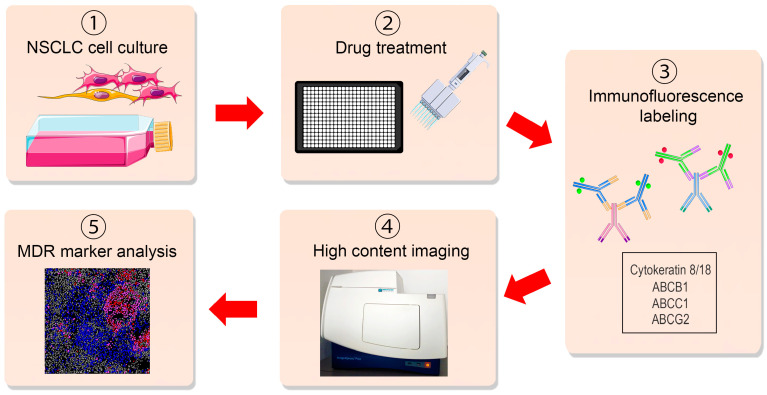
Schematic presentation of the immunofluorescence-based assay for high-throughput analysis of multidrug resistance markers in non-small cell lung carcinoma patient-derived cells. The key steps of the assay include (1) initial NSCLC culture establishment; (2) seeding the cells into black, clear bottom 384-well cell culture microplates and drug treatment; (3) fluorescent labeling of cells using anti-CK8/18, anti-ABCB1, anti-ABCC1, and anti-ABCG2 antibodies; (4) automated imaging using a high-throughput automated cell imaging system; (5) software-based analysis to score MDR marker-positive cancer cells, MDR marker-positive stromal cells, MDR marker-negative cancer cells, and MDR marker-negative stromal cells. This figure was created using images adapted from Servier Medical Art (Servier, smart.servier.com (accessed on 31 July 2023), licensed under a Creative Commons Attribution 3.0 Unported License).

Primary cultures often exhibit a heterogeneous composition, comprising both stromal cells and varying numbers of cancer cells, making it difficult to evaluate the effect of different drugs on the MDR phenotype across diverse cell populations [22]. To address this challenge, we created an immunofluorescence-based assay to assess MDR marker expression within patient-derived NSCLC cultures in an ex vivo context, serving as a valuable tool for pharmacological screening. The assay encompasses the drug treatment of cells, fluorescent immunolabeling, and subsequent automated cell imaging and analysis (Figure 1). The initial step involves the successful establishment of a primary NSCLC culture, as detailed in the Materials and Methods section. Subsequently, the cells are seeded into black, clear bottom 384-well cell culture microplates suitable for fluorescent cell labeling and subsequently treated with the drugs. Upon completion of the treatment regimen, the assay’s third step involves the fluorescent labeling of cells using anti-CK8/18, anti-ABCB1, anti-ABCC1, and anti-ABCG2 antibodies, as described in the Materials and Methods section. The anti-CK8/18 antibody cocktail distinguishes cancer cells in a mixed culture, while anti-ABCB1, anti-ABCC1, or anti-ABCG2 antibodies label MDR markers. Simultaneously, the cell nuclei are labeled with Hoechst 33342. The next step involves the imaging of the labeled cells using a high-throughput automated cell imaging system. Finally, the acquired images undergo software-based analysis, following the manufacturer’s instructions. This analysis serves to distinguish and quantify different cell populations within the primary culture: MDR marker-positive cancer cells, MDR marker-positive stromal cells, MDR marker-negative cancer cells, and MDR marker-negative stromal cells.

This assay is primarily designed to assess the increase in MDR marker-positive cells over time, rather than absolute MDR marker expression levels. Therefore, normalizing the MDR marker levels to the 0 h time point to account for baseline differences was not included, considering that the NSCLC patients in this study did not undergo chemotherapy or radiotherapy. It is important to recognize that the immunoassay has certain limitations, as it primarily identifies induced drug resistance through the presence of MDR marker-positive cells. Nonetheless, inherent resistance can still be assessed indirectly through sensitivity to different drugs.

### 3.2. Validation of Immunofluorescence Assay for the Assessment of MDR Markers in Mixed Cell Populations

**Figure 2:**
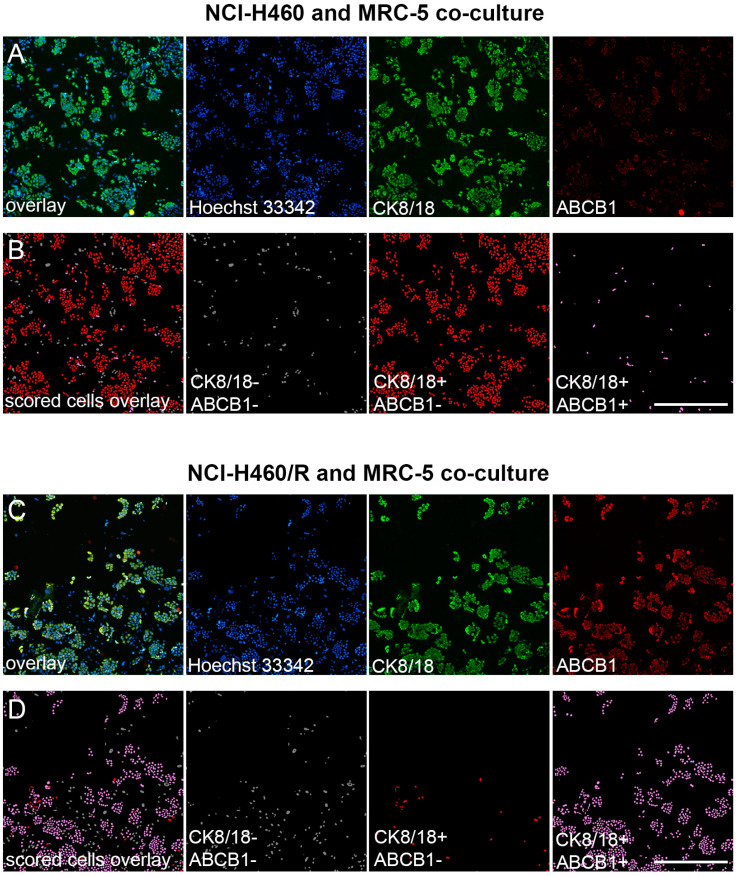
Image analysis of ABCB1-overexpressing cells in NCI-H460 and NCI-H460/R co-cultures with MRC-5 fibroblasts. (**A**) Fluorescence micrographs of the NCI-H460 and MRC-5 co-culture cultivated in 384-well cell culture microplates, fixed, and stained with Hoechst 33342 (blue), anti-CK8/18-Alexa Fluor 488 (green), and anti-ABCB1-Alexa Fluor 555 (red). The images were captured using ImageXpress Pico (Molecular Devices). Scale bar = 700 µm. (**B**) Cell scoring showing NCI-H460 population with low (CK8/18+/ABCB1−) and high ABCB1 expression (CK8/18+/ABCB1+), co-cultured with MRC-5 cells (CK8/18−/ABCB1−). The image analysis was performed using CellReporterXpress software’s Cell Scoring Analysis Protocol. (**C**) Fluorescence micrographs of the NCI-H460/R and MRC-5 co-culture cultivated in 384-well cell culture microplates, fixed, and stained with Hoechst 33342 (blue), anti-CK8/18-Alexa Fluor 488 (green), and anti-ABCB1-Alexa Fluor 555 (red). The images were captured using ImageXpress Pico (Molecular Devices). Scale bar = 700 µm. (**D**) Cell scoring showing NCI-H460/R population with low (CK8/18+/ABCB1−) and high ABCB1 expression (CK8/18+/ABCB1+), co-cultured with MRC-5 cells (CK8/18-/ABCB1−). The image analysis was performed using CellReporterXpress software’s Cell Scoring Analysis Protocol.

Our study demonstrates a new immunofluorescence-based assay for evaluating MDR markers in mixed cell populations. A pair of parental drug-sensitive (NCI-H460) and multidrug-resistant NSCLC cell lines with ABCB1 overexpression (NCI-H460/R) [23,24,25] were selected as a model to validate the assay (Figure 2). Furthermore, co-culturing cancer cell lines with normal MRC-5 fibroblasts, which lack ABCB1 expression, created a more realistic representation of the conditions found in primary NSCLC cultures. We employed a CK8/18 antibody cocktail to reliably distinguish the NSCLC cells, as this combination consistently identified epithelial cancer cells within mixed cell populations without staining human fibroblasts [12]. By employing a combination of anti-cytokeratin 8/18 and anti-ABCB1 antibodies, the cancer cells were reliably distinguished from the stromal cells, ensuring precise identification of drug-resistant cancer cells within mixed cultures. The simultaneous labeling of CK8/18 with the nuclear marker Hoechst 33342 enabled the discrimination and quantification of two distinct cell categories: CK8/18-negative cells, indicating MRC-5 cells, and CK8/18-positive cells, representing cancer cells (Figure 2). Additionally, the staining for ABCB1 enabled the recognition of cells expressing this MDR marker. The number of drug-sensitive cancer cells (CK8/18+/ABCB1−) and drug-resistant cancer cells (CK8/18+/ABCB1+) in each well was obtained through cell-scoring image analysis, as shown in Figure 2.

**Figure 3:**
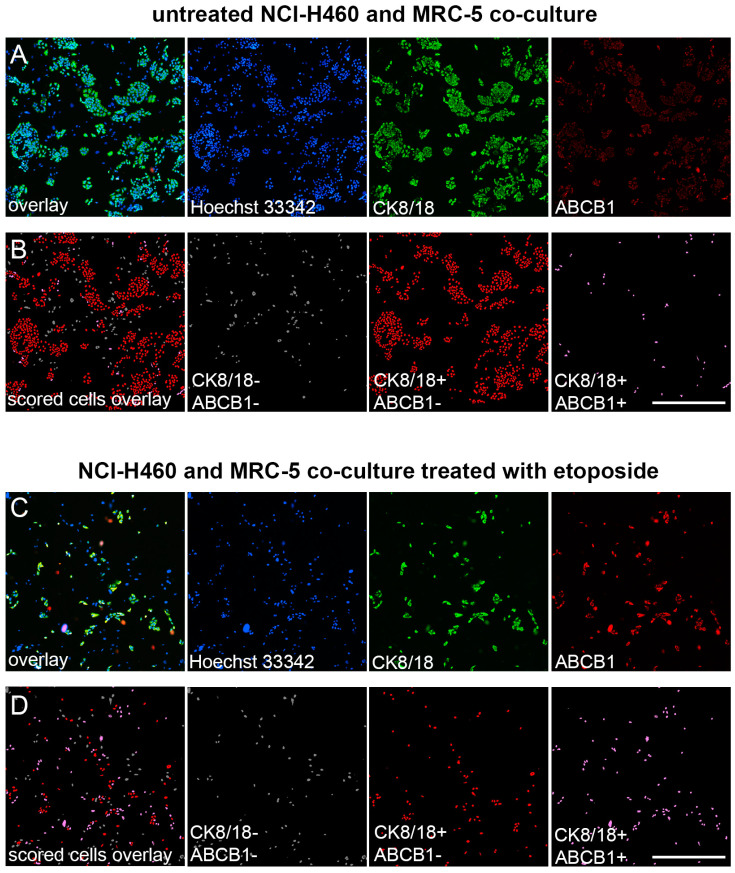
Image analysis of ABCB1-overexpressing cells in NCI-H460 and MRC-5 co-culture after etoposide treatment. (**A**) Fluorescence micrographs of the NCI-H460 and MRC-5 co-culture cultivated in 384-well cell culture microplates, fixed, and stained with Hoechst 33342 (blue), anti-CK8/18-Alexa Fluor 488 (green), and anti-ABCB1-Alexa Fluor 555 (red). The images were captured using ImageXpress Pico (Molecular Devices). Scale bar = 700 µm. (**B**) Cell scoring showing NCI-H460 population with low (CK8/18+/ABCB1−) and high ABCB1 expression (CK8/18+/ABCB1+), co-cultured with MRC-5 cells (CK8/18-/ABCB1−). The image analysis was performed using CellReporterXpress software’s Cell Scoring Analysis Protocol. (**C**) Fluorescence micrographs of the NCI-H460 and MRC-5 co-culture cultivated in 384-well cell culture microplates, treated with 10 µM etoposide for 24 h, fixed, and stained with Hoechst 33342 (blue), anti-CK8/18-Alexa Fluor 488 (green), and anti-ABCB1-Alexa Fluor 555 (red). The images were captured using ImageXpress Pico (Molecular Devices). Scale bar = 700 µm. (**D**) Cell scoring of etoposide effect showing NCI-H460 population with low (CK8/18+/ABCB1−) and high ABCB1 expression (CK8/18+/ABCB1+), co-cultured with MRC-5 cells (CK8/18−/ABCB1−). The image analysis was performed using CellReporterXpress software’s Cell Scoring Analysis Protocol.

The enrichment of the mixed cell culture with NCI-H460 cells overexpressing ABCB1 after treatment with 10 µM etoposide is illustrated in Figure 3.

The results demonstrated that the assay can accurately distinguish and quantify MDR markers in drug-sensitive and drug-resistant cancer cells within mixed cultures. This validation step highlights the assay’s utility in studying drug resistance mechanisms and supporting the development of personalized drug testing.

### 3.3. Assessment of MDR Marker Expression after Chemotherapeutics Treatment in NSCLC Cell Lines through Immunofluorescence Assay

Further validation of the fluorescence-based assay involved evaluating the effects of various chemotherapeutic agents on the expression of MDR markers in drug-sensitive and drug-resistant NSCLC cell lines. A wide range of drugs, including cisplatin, etoposide, paclitaxel, docetaxel, gemcitabine, vinorelbine, pemetrexed, and carboplatin, were tested either in single NCI-H460 and NCI-H460/R cultures, as well as in co-culture with MRC-5 cells. The IC_50_ values for each drug obtained in the single and co-cultures are shown in Table 1.

Cisplatin, etoposide, paclitaxel, docetaxel, gemcitabine, and vinorelbine showed efficacy within the range of applied concentrations in the NCI-H460 cells. The same drugs demonstrated selectivity towards cancer cells (NCI-H460), as their IC_50_ values were notably lower for these cells compared to normal cells (MRC-5). The NCI-H460/R cells showed resistance to several drugs, including etoposide, paclitaxel, and vinorelbine, as indicated by IC_50_ values that were significantly higher compared to the NCI-H460 cells. The resistance to vinorelbine was most pronounced, with the NCI-H460/R cells exhibiting a 27-fold resistance compared to the NCI-H460 cells. All the cells showed resistance towards pemetrexed and carboplatin within the applied concentration range. Among the drugs tested, docetaxel demonstrated potential in bypassing the resistance mechanism, as the IC_50_ value for the NCI-H460/R cells was in the effective range despite their resistance to several other drugs. Aside from cisplatin and gemcitabine, in the co-culture condition, the NCI-H460 cells generally exhibited similar sensitivity to most of the drugs compared to when they were cultured alone. The NCI-H460/R cells maintained their resistance in both monoculture and co-culture conditions.

Table 2 summarizes the effects of chemotherapy drugs on ABCB1 expression in single and co-cultures. The assay revealed that the expression of ABCB1 was significantly influenced by several chemotherapeutic agents, including cisplatin, etoposide, paclitaxel, docetaxel, and vinorelbine, in NCI-H460 cells. An increase in ABCB1 expression of at least 20% was considered biologically relevant. The drugs that increased ABCB1 expression are known substrates of ABCB1. Consequently, the increased expression of ABCB1 induced by these drugs likely contributed to the observed resistance in the NCI-H460/R cells, notably evident with etoposide, paclitaxel, and vinorelbine (Table 1). Interestingly, gemcitabine, pemetrexed, and carboplatin did not affect ABCB1 expression in these cells. When co-cultured with MRC-5 fibroblasts, the expression of ABCB1 in NCI-H460 cells was affected by etoposide, paclitaxel, docetaxel, and vinorelbine. The expression of ABCB1 in NCI-H460/R cells remained unchanged after treatment with chemotherapy drugs, both in single culture and in co-culture with fibroblasts. Similarly, MRC-5 cells showed no changes in ABCB1 expression in response to treatment, either in monocultures or co-cultures (Table 2).

**Figure 4:**
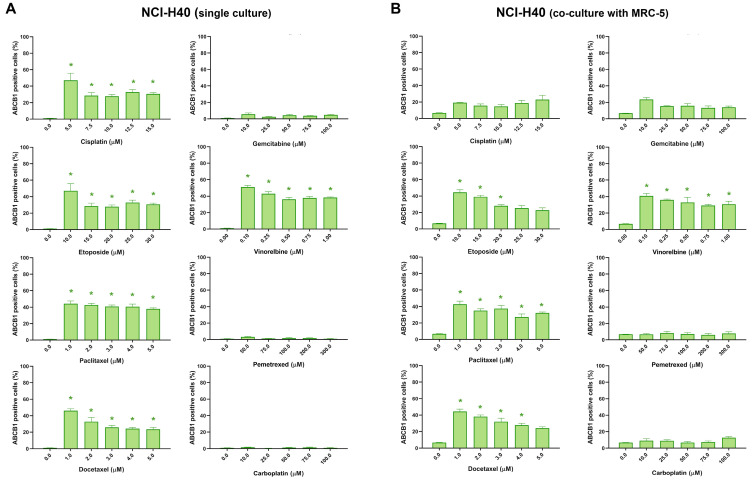
NCI-H460 cells expressing ABCB1 after treatment with chemotherapeutics. The NCI-H460 cells were treated in monoculture (**A**) and co-culture with MRC-5 fibroblasts (**B**). CK8/18 antibody was used to distinguish cancer cells from normal fibroblasts in co-culture. Graphs show the percentage of ABCB1-positive cells for each experimental condition. Data are presented as mean ± SEM (*n* = 4). A statistically significant difference between control and treated groups that showed an increase in ABCB1 expression of at least 20% is indicated as * (*p* < 0.05).

ABCB1 expression in NCI-H460 cells after treatment with five increasing concentrations of chemotherapeutics is shown in Figure 4. The expression profile of ABCB1 in NCI-H460 cells in co-cultures is similar to that recorded in monoculture, indicating that the assay is capable of distinguishing and evaluating cancer cells in a mixed culture. This demonstrates the feasibility of using this assay to identify and analyze cancer cells in a complex environment. The presence of fibroblasts appears to have no effect on the development of resistance in NCI-H460 cells after treatment with chemotherapeutic drugs.

### 3.4. Assessment of MDR Marker Expression after Chemotherapeutics Treatment in Patient-Derived NSCLC Cultures through Immunofluorescence Assay

After validating the assay’s efficacy using established cell lines, we tested the assay’s ability to distinguish cancer from stromal cells and assess their MDR profile in five patient-derived NSCLC cultures. The primary cultures were treated with cisplatin, etoposide, paclitaxel, docetaxel, gemcitabine, vinorelbine, pemetrexed, and carboplatin. The IC_50_ values for each drug obtained in the NSCLC cultures using the assay are shown in Table 3.

The patient-derived cultures showed a range of drug sensitivity and resistance patterns, demonstrating variability in their responses to applied chemotherapeutics. Although the drug responses varied widely among the different cultures, vinorelbine displayed the highest efficacy. Compared to the cell lines, the patient-derived cultures demonstrated significantly higher sensitivity to gemcitabine, pemetrexed, and carboplatin. Cisplatin, etoposide, docetaxel, gemcitabine, and pemetrexed showed selective cytotoxicity towards CK8/18+ cells in some cultures. Carboplatin and vinorelbine exhibited higher sensitivity towards non-cancer cells in all the tested cultures, implying a potential for greater cytotoxic effects on normal tissues. Considering this, the assay’s ability to detect differences in drug cytotoxicity, including potential side effects on normal cells, holds promise for optimizing personalized cancer treatments.

When evaluating drug responses, we found that NCI-H460 cells, when co-cultured with fibroblasts, exhibited relatively lower resistance compared to CK8/18+ cells from primary cultures. In fact, the cancer cells in primary cultures demonstrated drug responses more similar to NCI-H460/R cells when co-cultured with fibroblasts. Overall, patient-derived cultures displayed a broader range of response patterns compared to cell line models, highlighting their inherent diversity.

Table 4 provides a summary of the effects of cisplatin, etoposide, paclitaxel, docetaxel, gemcitabine, vinorelbine, pemetrexed, and carboplatin on the expression of ABCB1, ABCC1, and ABCG2 in cancer cells (CK8/18+) within the primary NSCLC cultures. The change in ABCB1 expression in CK8/18+ cells after treatment with each chemotherapy drug was patient-dependent, with paclitaxel causing an increase in the expression of this MDR marker in all the primary cultures. Paclitaxel also increased the expression of ABCC1 (aside from culture TR58) and ABCG2 in all the CK8/18+ cells. The weakest effect on the expression of MDR markers was observed in the pemetrexed treatment (Table 4). It is noteworthy that, in the majority of tested NSCLC cultures, the treatments had no significant impact on the expression of MDR markers in non-cancer cells (CK8/18−). However, in sample TR58, elevated ABCC1 expression was observed in CK8/18− cells following treatment with paclitaxel, docetaxel, and gemcitabine, while increased ABCG2 expression was detected after cisplatin administration. Sample TR65 had elevated ABCG2 expression in CK8/18− cells after the administration of gemcitabine.

**Figure 5:**
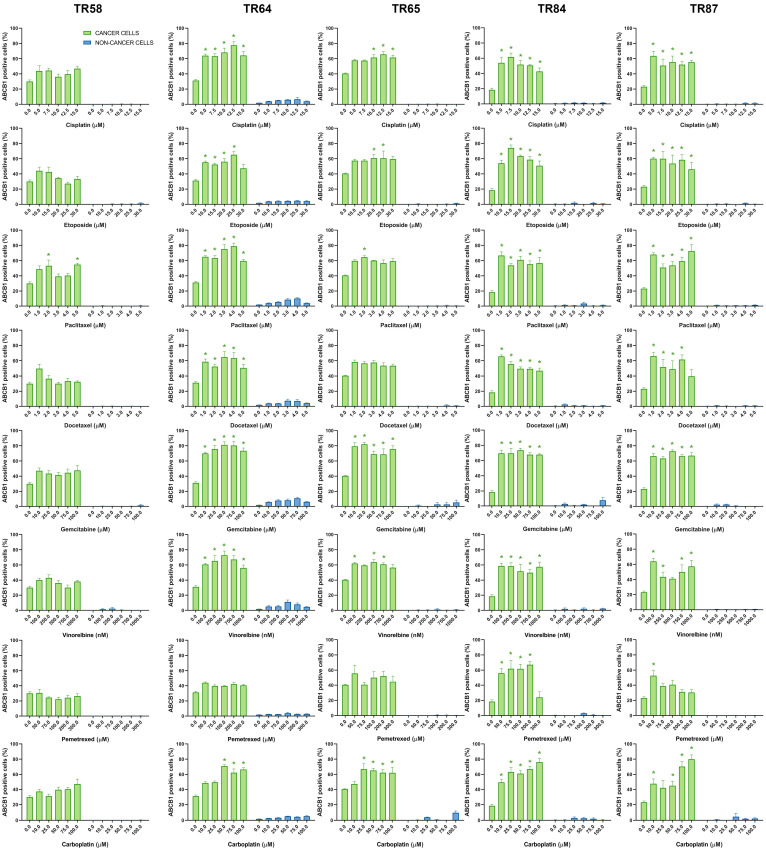
Patient-derived NSCLC cultures expressing ABCB1 after treatment with chemotherapeutics. CK8/18 antibody was used to distinguish cancer cells from non-cancer cells in mixed culture. Graphs show the percentage of ABCB1-positive cells for each experimental condition. Data are presented as mean ± SEM (*n* = 4). A statistically significant difference between control and treated groups that showed an increase in ABCB1 expression of at least 20% is indicated as * (*p* < 0.05).

**Figure 6:**
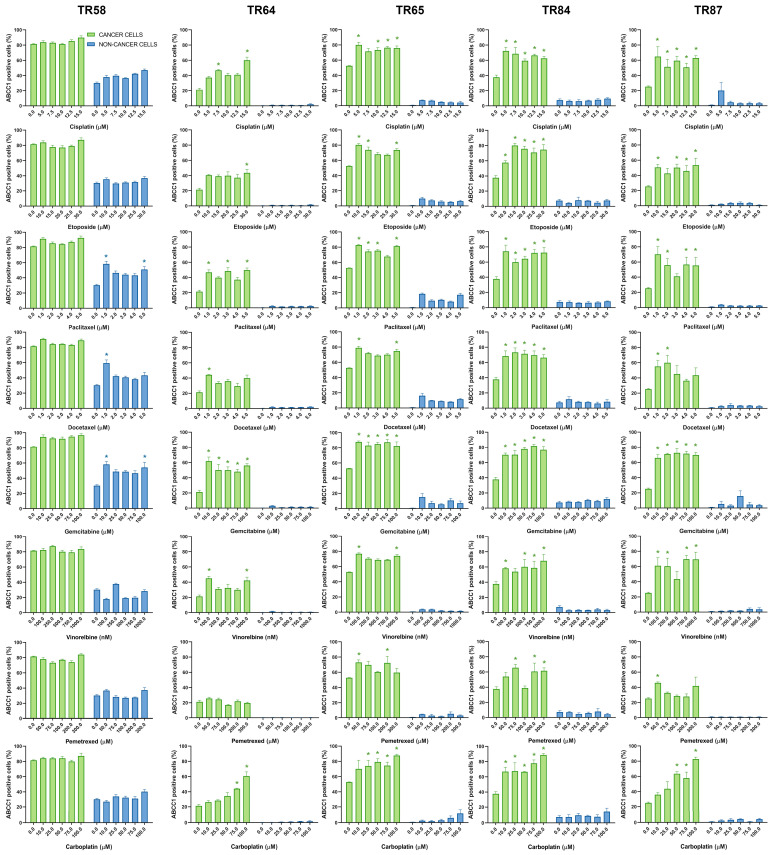
Patient-derived NSCLC cultures expressing ABCC1 after treatment with chemotherapeutics. CK8/18 antibody was used to distinguish cancer cells from non-cancer cells in mixed culture. Graphs show the percentage of ABCC1-positive cells for each experimental condition. Data are presented as mean ± SEM (*n* = 4). A statistically significant difference between control and treated groups that showed an increase in ABCC1 expression of at least 20% is indicated as * (*p* < 0.05).

**Figure 7:**
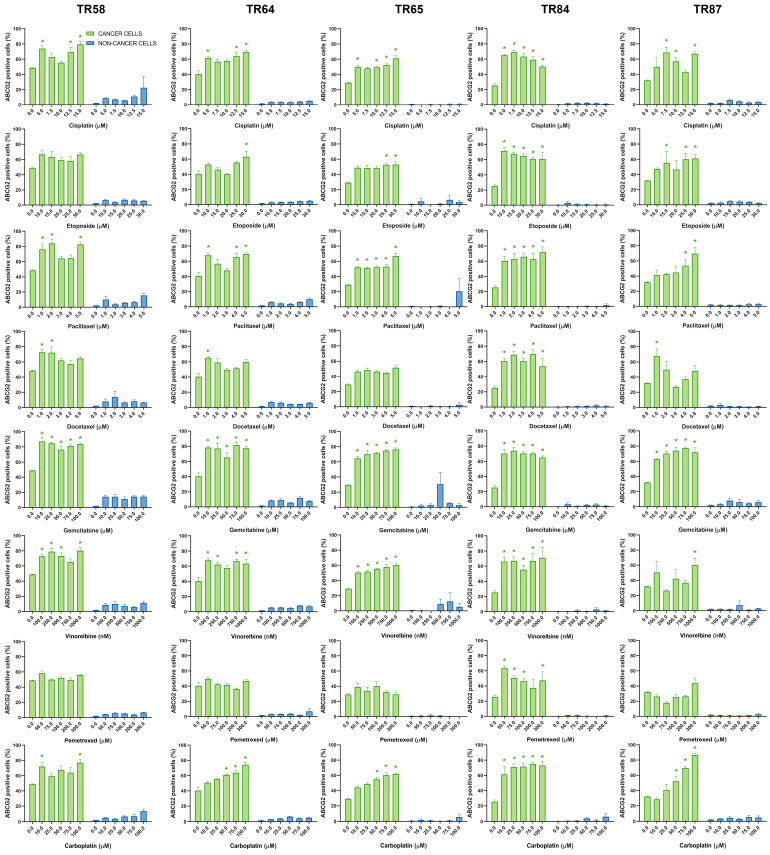
Patient-derived NSCLC cultures expressing ABCG2 after treatment with chemotherapeutics. CK8/18 antibody was used to distinguish cancer cells from non-cancer cells in mixed culture. Graphs show the percentage of ABCG2-positive cells for each experimental condition. Data are presented as mean ± SEM (*n* = 4). A statistically significant difference between control and treated groups that showed an increase in ABCG2 expression of at least 20% is indicated as * (*p* < 0.05).

The changes in ABCB1, ABCC1, and ABCG2 expression in the primary NSCLC cultures after treatment with five increasing concentrations of chemotherapeutics are shown in Figure 5, Figure 6 and Figure 7, respectively.

The findings regarding the differential impact of chemotherapeutic agents on the expression of MDR markers highlight the importance of tailoring treatment strategies to individual patients. For instance, the increase in ABCB1 expression in all the primary NSCLC cultures after the application of paclitaxel suggests that using this drug may lead to the development of the MDR phenotype. This can make paclitaxel less effective, as well as other structurally and functionally unrelated drugs. Conversely, pemetrexed only sporadically induces MDR markers. The obtained results demonstrate the utility of the assay in analyzing the expression profiles of ABCB1, ABCC1, and ABCG2 in NSCLC patient samples ex vivo. By discriminating between cancer and non-cancer cells, the assay provides valuable insights into drug effects on MDR markers specific to different cell populations, highlighting the assay’s potential as a tool for drug discovery and personalized medicine in NSCLC.

To further underscore the clinical relevance of our immunoassay, we evaluated the response of NSCLC patient-derived cultures to tyrosine kinase inhibitor (TKI) to demonstrate the applicability of the assay in assessing the effects of TKI treatments for NSCLC patients. NSCLC cultures were treated with gefitinib, a well-known EGFR inhibitor, to evaluate its influence on the ABCC1 expression.

CK8/18+ cancer cells in sample TR65 exhibited the highest sensitivity to gefitinib, with an IC_50_ value of 148.5 nM, indicating a relatively favorable response to the drug (Supplementary Table S1). On the contrary, the CK8/18+ cancer cells in other cultures showed reduced sensitivity to gefitinib, suggesting inherent resistance to the drug. In the majority of the cultures, non-cancer cells exhibited resistance to gefitinib, except for sample TR87, which showed an IC_50_ value of 188.4 nM. It is also important to note that sample TR64 harbors the EGFR L858R mutation, a well-known driver mutation in NSCLC that normally leads to enhanced sensitivity to EGFR TKIs such as gefitinib [26]. The lack of response of TR64 to clinically relevant concentrations of gefitinib indicates the complexity of TKI responses in NSCLC patients [26,27]. The heterogeneity in gefitinib responses among NSCLC cultures detected in our immunoassay highlights the importance of personalized TKI treatment approaches based on individual patient profiles.

Supplementary Table S2 provides a summary of the effects of gefitinib on the expression of ABCC1 in cancer (CK8/18+) and non-cancer (CK8/18−) cells within primary NSCLC cultures. In samples TR65 and TR84, an increase in ABCC1-expressing cancer cells was observed, while non-cancer cells were unaffected. TR58, TR64, and TR87 displayed no significant change in ABCC1 expression for both cancer and non-cancer cells after gefitinib treatment, emphasizing the diversity in drug response across various NSCLC cultures, which holds significance for personalized therapeutic strategies.

The changes in ABCC1 expression in the primary NSCLC cultures after treatment with five increasing concentrations of gefitinib are shown in Supplementary Figure S1.

Variability in drug responses among different cellular models, including cell lines and patient-derived NSCLC cultures, underscores the complex landscape of personalized medicine. Our study revealed a spectrum of drug sensitivity and resistance patterns, emphasizing the need for tailored treatment approaches that consider individual patient profiles. This approach enables the identification of specific drugs with enhanced effectiveness in particular cases while mitigating the risk of adverse effects on healthy tissues. Consequently, this methodology shows potential in optimizing personalized cancer treatments and advancing precision medicine.

The developed immunofluorescence-based assay supports the growing interest in functional diagnostics, which evaluates the functional responses of cells to drugs and stimuli, complementing genetic profiling. By combining ex vivo pharmacological screening with diagnostics, the assay could help clinicians determine the best treatment for NSCLC patients based on their individual profiles. This approach has the potential to enhance treatment efficacy while minimizing adverse effects. With appropriate modifications and adaptations, this immunofluorescence-based functional assay could potentially be used for other cancer types to assess the response to therapy and evaluate the development of drug resistance. In addition to its current application in assessing ABCB1, ABCC1, and ABCG2 as markers of MDR, the assay could be further adapted to evaluate other MDR mechanisms beyond ABC transporters. Future adaptations may focus on investigating the impact of diverse mutational profiles on resistance mechanisms, such as EGFR and ROS mutations, and exploring their connection to TKI response in NSCLC.

While this study primarily serves as a methodological foundation, we recognize the significance of translating our findings into clinical applications. The observed drug testing profiles offer potential for future investigations that aim to correlate these profiles with clinical treatment outcomes and patient prognosis and demonstrate the practical implications of the methodology presented in this paper.

## 4. Conclusions

In summary, the described immunofluorescence-based assay provides a robust platform for evaluating MDR marker expression in complex cell populations, including primary NSCLC cultures. The results offer valuable insights into the differential impact of anticancer drugs on MDR markers and highlight the potential for personalized therapy selection based on individual patient profiles. While this assay offers a number of advantages, it has limitations. Short-term primary cultures may not accurately reflect the full therapeutic cycle, making it challenging to determine if MDR marker induction is temporary or permanent. Although the assay generally provides effective segmentation for individual nuclei and cells, it may encounter occasional challenges in cases of fully overlapped nuclei, which should be considered in practical applications. Nevertheless, this assay represents a promising tool in the pursuit of more effective treatments for NSCLC patients and may have broader applications in cancer research and drug development. A significant contribution to the development of the assay would be its optimization for the simultaneous application of therapeutics, a research direction we aim to explore in the future.

## Figures and Tables

**Table 1:** IC_50_ values of chemotherapeutics in NCI-H460, NCI-H460/R, and MRC-5 cells.

Cell Lines	IC_50_ (µM)							
	Cisplatin	Etoposide	Paclitaxel	Docetaxel	Gemcitabine	Vinorelbine	Pemetrexed	Carboplatin
NCI-H460	5.705	4.809	0.3808	0.5541	41.565	0.03656	>300	>100
NCI-H460 (co-culture with MRC-5)	9.791	6.074	0.3391	0.6416	34.588	0.03332	>300	>100
NCI-H460/R	5.334	7.922	>5	0.7041	33.588	0.9902	>300	>100
NCI-H460/R (co-culture with MRC-5)	5.599	7.500	>5	0.8008	29.388	0.8385	>300	>100
MRC-5	14.682 ^s^	>30 ^s^	>5 ^s^	>5 ^s^	>100 ^s^	>1 ^s^	>300	>100
MRC-5 (co-culture with NCI-H460)	>15 ^s^	>30 ^s^	>5 ^s^	>5 ^s^	>100 ^s^	0.4773 ^s^	>300	>100
MRC-5 (co-culture with NCI-H460/R)	>15 ^s^	>30 ^s^	>5	>5 ^s^	>100 ^s^	>1 ^s^	>300	>100

^s^ Selectivity towards sensitive NCI-H460 cells.

**Table 2:** Effect of chemotherapeutics on ABCB1 expression in NCI-H460, NCI-H460/R, and MRC-5 cells.

Cell Lines	ABCB1 Expression *							
	Cisplatin	Etoposide	Paclitaxel	Docetaxel	Gemcitabine	Vinorelbine	Pemetrexed	Carboplatin
NCI-H460	increase	increase	increase	increase	no change	increase	no change	no change
NCI-H460 (co-culture with MRC-5)	no change	increase	increase	increase	no change	increase	no change	no change
NCI-H460/R	no change	no change	no change	no change	no change	no change	no change	no change
NCI-H460/R (co-culture with MRC-5)	no change	no change	no change	no change	no change	no change	no change	no change
MRC-5	no change	no change	no change	no change	no change	no change	no change	no change
MRC-5 (co-culture with NCI-H460)	no change	no change	no change	no change	no change	no change	no change	no change
MRC-5 (co-culture with NCI-H460/R)	no change	no change	no change	no change	no change	no change	no change	no change

* An increase in ABCB1 expression of at least 20% at a minimum of one concentration is shown in the table.

**Table 3:** IC_50_ values of chemotherapeutics in patient-derived NSCLC cultures.

NSCLC Cultures	IC_50_ (µM)							
	Cisplatin	Etoposide	Paclitaxel	Docetaxel	Gemcitabine	Vinorelbine	Pemetrexed	Carboplatin
Cancer cells (CK8/18+)								
TR58	5.780	7.131	1.180	1.312	2.550	0.1291	180.739	60.842
TR64	6.067	11.919	1.354	1.560	4.924	0.1716	251.855	38.982
TR65	14.636	25.393	1.180	>5	4.912	>1	231.473	22.492
TR84	2.112	2.983	2.568	1.850	2.683	0.1763	>300	8.147
TR87	4.821	12.980	4.787	>5	3.407	>1	>300	43.253
Non-cancer cells (CK8/18-)								
TR58	5.104	6.490	1.399	1.594	3.074 ^s^	0.1601	>300 ^s^	27.334
TR64	8.255 ^s^	18.133 ^s^	1.953	2.725 ^s^	7.063 ^s^	0.1840	>300 ^s^	12.755
TR65	1.205	2.076	1.399	0.4610	0.3511	0.03281	25.177	2.161
TR84	0.7891	1.013	0.312	0.1819	0.4909	0.03387	>300	1.280
TR87	3.381	10.205	1.866	3.051	1.128	0.3606	>300	22.921

^s^ Selectivity towards cancer cells.

**Table 4:** Effect of chemotherapeutics on ABCB1, ABCC1, and ABCG2 expression in patient-derived NSCLC cultures.

NSCLC Cultures Cancer Cells (CK8/18+)	Cisplatin	Etoposide	Paclitaxel	Docetaxel	Gemcitabine	Vinorelbine	Pemetrexed	Carboplatin
	ABCB1 expression *							
TR58	no change	no change	increase	no change	no change	no change	no change	no change
TR64	increase	increase	increase	increase	increase	increase	no change	increase
TR65	increase	increase	increase	no change	increase	increase	no change	increase
TR84	increase	increase	increase	increase	increase	increase	increase	increase
TR87	increase	increase	increase	increase	increase	increase	increase	increase
	ABCC1 expression *							
TR58	no change	no change	no change	no change	no change	no change	no change	no change
TR64	increase	increase	increase	increase	increase	increase	no change	increase
TR65	increase	increase	increase	increase	increase	increase	increase	increase
TR84	increase	increase	increase	increase	increase	increase	increase	increase
TR87	increase	increase	increase	increase	increase	increase	increase	increase
	ABCG2 expression *							
TR58	increase	no change	increase	increase	increase	increase	no change	increase
TR64	increase	increase	increase	increase	increase	increase	no change	increase
TR65	increase	increase	increase	no change	increase	increase	no change	increase
TR84	increase	increase	increase	increase	increase	increase	increase	increase
TR87	increase	increase	increase	increase	increase	increase	no change	increase

* An increase in ABCB1, ABCC1, or ABCG2 expression of at least 20% at a minimum of one concentration is shown in the table.

## Data Availability

The data presented in this study are available from the corresponding author upon reasonable request.

## Supplementary Materials

The following supporting information can be downloaded at: https://www.mdpi.com/article/10.3390/diagnostics13243617/s1, Figure S1: Patient-derived NSCLC cultures expressing ABCC1 after treatment with tyrosine kinase inhibitor gefitinib; Table S1: IC_50_ values of tyrosine kinase inhibitor gefitinib in patient-derived NSCLC cultures; Table S2: Effect of tyrosine kinase inhibitor gefitinib on ABCC1 expression in patient-derived NSCLC cultures.
