# Supplementary material for: Immunofluorescence-Based Assay for High-Throughput Analysis of Multidrug Resistance Markers in Non-Small Cell Lung Carcinoma Patient-Derived Cells
Source: Diagnostics (Basel). 2023 Dec 7;13(24):3617. doi: 10.3390/diagnostics13243617 (PMC10743086; doi:10.3390/diagnostics13243617)
Supplement: Supplementary file 1 [file diagnostics-13-03617-s001.zip › Supplementary Material.pdf]

Supplementary Material

Table S1. IC_{50} values of tyrosine kinase inhibitor gefitinib in patient-derived NSCLC cultures.

NSCLC cultures	gefitinib IC_{50} (nM)	
	Cancer cells (CK8/18+)	Non-cancer cells (CK8/18-)
TR58	>400	>400
TR64	>400	>400
TR65	148.5[s]	>400
TR84	>400	>400
TR87	>400	188.4

[s] selectivity towards cancer cells

Table S2. Effect of tyrosine kinase inhibitor gefitinib on ABCC1 expression in patient-derived NSCLC cultures.

	ABCC1 expression*	
NSCLC cultures	Cancer cells (CK8/18+)	Non-cancer cells (CK8/18-)
TR58	no change	no change
TR64	no change	no change
TR65	increase	no change
TR84	increase	no change
TR87	no change	no change

*an increase in ABCC1 expression of at least 20% at a minimum of one concentration is shown in the Table.

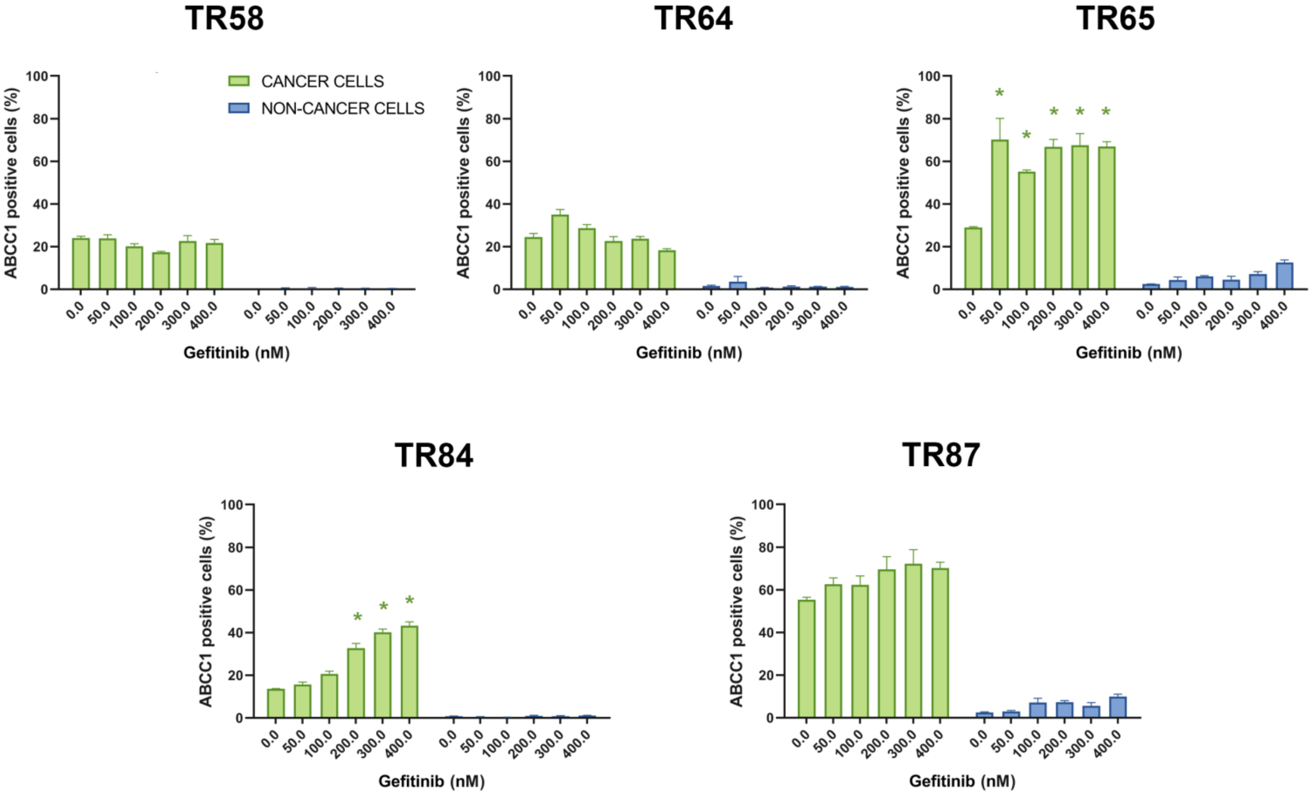

Figure S1. Patient-derived NSCLC cultures expressing ABCC1 after treatment with tyrosine kinase inhibitor gefitinib. CK8/18 antibody was used to distinguish cancer cells from non-cancer cells in mixed culture. Graphs show the percentage of ABCC1-positive cells for each experimental condition. Data are presented as mean ± SEM ($n = 4$). A statistically significant difference between control and treated groups that showed an increase in ABCC1 expression of at least 20% is indicated as * ($p < 0.05$).
